# β-Sitosterol Contributes in the Resistance to Invasion and Survival of *Brucella abortus* 544 within RAW264.7 Cells, and Cytokine Production with Reduced Susceptibility to Infection in BALB/c Mice

**DOI:** 10.4014/jmb.1909.09052

**Published:** 2019-12-30

**Authors:** Alisha Wehdnesday Bernardo Reyes, Lauren Togonon Arayan, Tran Xuan Ngoc Huy, Son Hai Vu, Wongi Min, Jin Hur, Suk Kim

**Affiliations:** 1 Institute of Animal Medicine, College of Veterinary Medicine, Gyeongsang National University, Jinju 52828, Republic of Korea; 2 Veterinary Public Health, College of Veterinary Medicine, Chonbuk National University, Iksan 54596, Republic of Korea

**Keywords:** Brucella abortus, β-sitosterol, macrophage, internalization, cytokine

## Abstract

We previously identified β-sitosterol (BS) as one of the most abundant compounds found in Korean red ginseng oil. BS is a widely prevalent vegetable-derived phytosterol with many known health benefits. Here, we investigated the efficacy of BS against *Brucella* (*B.*) *abortus* infection. BS showed no effect on bacterial growth but attenuated internalization, intracellular survival and MAPKs-linked intracellular signaling in RAW264.7 cells. BS treatment in cells is also associated with increased nitrite concentration during infection at 24 h. Slightly enhanced resistance to *B. abortus* infection was observed in mice orally given BS, which could be mediated by induced production of proinflammatory cytokines. Taken together, our study demonstrates the contribution of BS treatment against *B. abortus* infection although further investigation is encouraged to maximize its beneficial effects against intracellular infection.

## Introduction

*Brucella abortus* is a highly pathogenic zoonotic agent, a common source of human infection and with 500,000 reported cases annually, brucellosis stands first in the list of zoonotic bacterial diseases [1, 2]. Fever is one of the most common symptoms in human but the disease also increases the risk of spontaneous abortion, premature delivery, miscarriage and intrauterine infection with fetal death accompanied with malaise, fatigue and arthritis [2]. The etiologic agent has developed a stealthy strategy that allows it to circumvent strong innate immune system activation, withstand the direct action of complement and other bactericidal substances, evade the action of professional phagocytes, and maintain the host cells alive to establish long lasting infections [3]. There is no current available specific diagnostic test to identify *Brucella* with consistent false negativity obtained using serological tests at the early days of infection [2]. Although mortality rate is low, treatment of human brucellosis is still controversial with no prophylaxis [1]. Current standard treatment regimens for brucellosis include a combination of tetracycline, trimethoprim-sulfamethoxazole, aminoglycosides, rifampicin, quinolones, chloramphenicol, doxycycline and streptomycin but a higher incidence of failure or relapse still occurs in monotherapy [4]. Furthermore, an increasing problem of antibiotic resistance poses a serious global threat to human, animal and environmental health [5].

In a study done by Nath *et al*. [6], the blood biochemical metabolites of crossbred cattle suffering from brucellosis showed increased serum cholesterol level in comparison to healthy cattle, and Ali [7] documented abnormally high serum concentration of total cholesterol in brucellosis patients than in healthy control group. Lin *et al*. [8] reported that the role of cholesterol in pathogen-host interactions contributed to promote the survival of pathogen and its virulence delivery into host. On the other hand, β-sitosterol (BS) is a compound structurally related to cholesterol that is slowly absorbed in the intestinal tract, hence can interfere in the absorption of cholesterol and its rise in serum [9]. BS is a natural micronutrient found in numerous plants and considered as one of the most prevalent vegetable-derived phytosterols in the diet proven to be a safe, non-toxic, effective nutritional supplement with potential health benefits in many diverse applications including antibacterial and antiviral activities, modulation of immune function, inflammation and pain levels by controlling the production of inflammatory cytokines, and inhibitor of arthritis, ulcer and cancer [9, 10]. Furthermore, we previously reported that BS is one of the abundant compounds identified in the phytochemical analysis of Korean red ginseng oil which could be attributed to its immunomodulatory activity [12]. Given the reported beneficial properties of BS, we investigate its effect against *B. abortus* infection using a murine macrophage cell line and a murine model in the context of phagocytosis and intracellular bacterial survival, and immune modulation and control of bacterial proliferation, respectively, for finding a viable alternative approach that is natural, safe and effective for the treatment and control of brucellosis.

## Materials and Methods

### β-Sitosterol (BS) Preparation

BS was purchased from Sigma Chemical Co. (molecular weight 414.71 g/mol, USA), dissolved in absolute ethanol (100 mM) and diluted in sterile phosphate buffered saline (PBS) solution (pH 7.4) containing 0.1% bovine serum albumin (BSA) (GenDEPOT, USA).

### Bacterial Strain and Cultivation

The standard wild-type *B. abortus* 544 (ATCC 23448) was maintained in *Brucella* broth (Becton Dickin, USA) at 37°C for 2 days with vigorous shaking. Serial dilutions of a sample were plated on Brucella agar incubated at 37°C for 3 days to determine colony forming units (CFUs).

### Cell Culture and Cultivation

The RAW264.7 macrophage cell line (ATCC TIB7-1, USA) was maintained in RPMI 1640 medium containing 10% heat-inactivated fetal bovine serum (FBS), 100 U/ml penicillin and 100 μg/ml streptomycin (Invitrogen, USA) in a 5% CO_2_ atmosphere at 37°C. Prior to infection, the medium was changed to fresh medium without antibiotics.

### Cytotoxicity Assay

RAW264.7 cells (1 × 10^5^ cells/well) were seeded into a 96-well tissue culture plate and cultured overnight. Cells were then treated with different concentrations of BS (0, 10, 20, 50, 100, 200, 500 μM) for 48 h in fresh medium. Viability of cells was assessed using MTT assay as previously described [11]. A 0.1% ethanol and 0.1% BSA in appropriate medium was used as control in all experiments. The highest non-cytotoxic concentration of BS was used in the succeeding experiments.

### Bactericidal Assay

*B. abortus* (2 × 10^4^ CFU/well) were incubated into different concentrations of BS (0, 10, 50, 100, 500 μM) in a 96-well plate for 0, 2, 24, and 48 h in PBS. Each sample was serially diluted using PBS and plated on Brucella agar to determine the direct effect of BS on *B. abortus* growth. Bacterial survival rates were determined as a percentage of survival rate of BS-treated sample relative to control sample which was set to 100%.

### Nitrite Assay

Preparation of RAW264.7 cells was similar with cytotoxicity assay. The cells were pre-treated with BS (20 μM) for 4 h and then washed prior to infection. *B. abortus* at a multiplicity of infection (MOI) of 100 were deposited onto cells, centrifuged at 150 ×*g* for 10 min and then incubated for 1 h at 37°C in a 5% CO_2_ atmosphere. The cells were washed and incubated in fresh medium containing gentamicin (30 μg/ml) with or without BS (20 μM) for 2, 24, and 48 h. Nitrite accumulation was measured as an indicator of nitric oxide (NO) production based on Griess reaction (Promega Corporation, USA) according to manufacturer’s instruction.

### Infection Assay

To determine the efficiency of bacterial uptake, cells were prepared similar to cytotoxicity assay and then pre-treated with BS (20 μM) for 4 h prior to infection. Bacteria were deposited onto cells at MOI of 100, centrifuged for 10 min and then incubated for 0 and 30 min at 37°C in a 5% CO_2_ atmosphere. After incubation, the cells were washed three times, incubated in fresh medium with gentamicin for 30 min and then lysed with distilled water. To determine the efficiency of bacterial intracellular growth, cells were prepared as that of the invasion assay but the cells were infected first for 1 h prior to incubation in fresh medium containing gentamicin with BS (20 μM) for 2, 24, and 48 h. After incubation, cells were washed and then lysed with distilled water. Each sample was serially diluted and then plated onto Brucella agar to determine the number of viable bacteria by counting CFUs as previously described [11].

### Immunoblot Analysis

RAW264.7 cells (1 × 10^6^ cells/well) were seeded into a 6-well tissue culture plate and cultured overnight. Pre-treatment and infection of cells were the same as in invasion assay. At 30 min pi, cell lysates were collected, protein concentrations were determined using Bradford protein assay and were boiled for 5 min in 2x Laemmli sample buffer (Bio-Rad Lab. Inc., USA). Immunoblot assay was performed with slight modifications as we previously described [11]. Briefly, membranes were incubated with phospo-specific antibodies against ERK, JNK and p38α (1:250, Cell Signaling Technology, Inc., USA) in 5% bovine serum albumin (GenDEPOT, USA) at 4°C overnight. Incubation with secondary antibody was done using peroxidase-conjugated anti-rabbit IgG (Thermo Scientific, USA; 1:1,000 dilution) at room temperature for 1 h. β-actin antibody was used as a loading control. Membranes were exposed to a Molecular Imager ChemiDoc XRS+ system machine (Bio-Rad Lab.). NHI ImageJ software (USA) was used to quantify the immunoblots.

### In vivo Experiment

Eight-week old pathogen-free female BALB/c mice (Samtako Bio Co. Ltd., Korea) were acclimatized for one week and assigned randomly into four groups of five mice each. The animals were orally given with 100 μl of BS (20 μM) or vehicle (0.1% ethanol and 0.1% BSA in PBS) using a gavage needle for three days prior to infection until 14 days post-infection (pi). The groups were subdivided into infected and non-infected groups. The infected groups were intraperitoneally infected with *B. abortus* (2 × 10^4^ CFU in 100 μl PBS) and the blood was collected via tail vein at 3 days post-infection (pi). The mice were sacrificed, blood was collected from the heart and the spleens were collected and weighed. A part of each spleen was homogenized and serially diluted in PBS. The samples were then plated onto Brucella agar and incubated at 37°C for 3 days to determine the number of CFUs in each spleen. The aforementioned procedures were in compliance with established federal guidelines and institutional policies by the Animal Ethical Committee of Chonbuk National University (Authorization Number CBNU-2019-53).

### Cytometric Bead Array (CBA)

Serum samples were collected and processed to measure the level of different cytokines involved in the outcome of *Brucella* infection such as IL-12p70, TNF, IFN-γ, MCP-1, IL-10, and IL-6 using a Cytometric Bead Array (CBA) mouse inflammation kit (BD Biosciences, USA) according to manufacturer’s instruction. The samples were acquired on a FACSCalibur flow cytometer (BD Biosciences) and analyzed using BD CellQuest software.

### Enzyme-Linked Immunosorbent Assay (ELISA)

Serum alanine aminotransferase 1 (ALT) concentration was determined using an ALT (Mouse) ELISA kit (BioVision Inc., USA) to determine liver damage or injury during the course of BS treatment while IL-1β was measured using IL-1 beta Mouse SimpleStep ELISA kit (Abcam, USA) according to manufacturer’s instructions.

### Statistical Analysis

The data are expressed as the mean ± standard deviation (SD) and comparisons between groups were analyzed using Student’s *t-*test. *p* < 0.05 was considered as statistically significant.

## Results

### Effect of BS on RAW264.7 Cell Viability, *B. abortus* Growth and Nitrite Production

**Fig. 1:**
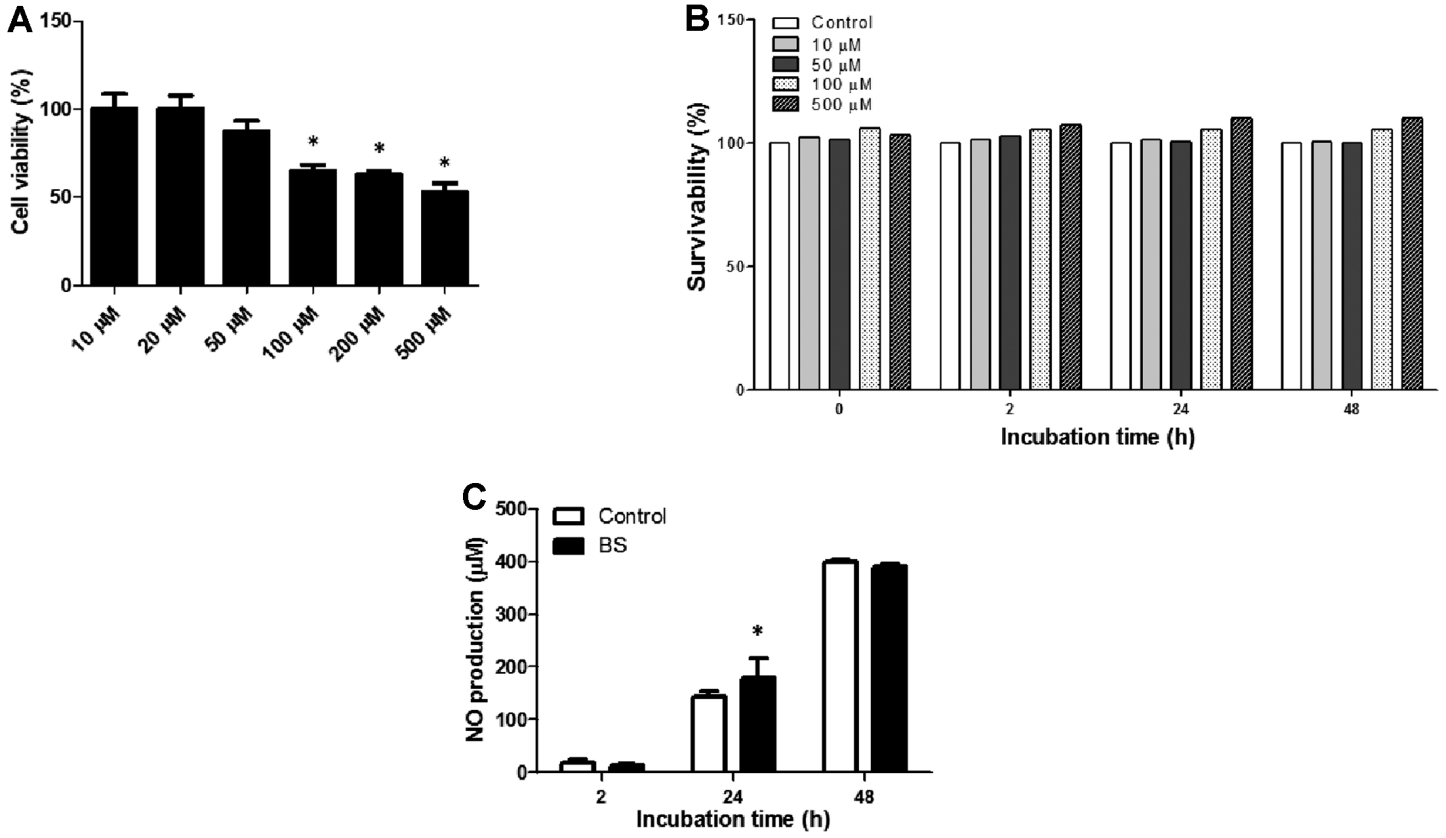
Effect of BS on cell viability, bacterial survivability and nitrite production. RAW264.7 cells were incubated with different concentrations of BS for 48 h and (**A**) cell viability was evaluated using MTT assay. *B. abortus* was incubated with different concentrations of BS for 0, 2, 24, and 48 h, and (**B**) bacterial survival rates were determined. Cells were pre-incubated with or without BS and then infected with *B. abortus* for 1 h, and (**C**) nitrite accumulation was measured after 2, 24, and 48 h by Griess reaction. The data are presented as the means ± SD of triplicate experiments. *p < 0.05 *vs.* control group.

In the cytotoxicity assay, a reduced OD value was observed at concentrations 50, 100, 200, and 500 μM. The highest non-cytotoxic concentration of BS (20 μM) in which the viability of RAW264.7 cells remained 100% was used in the succeeding experiments (Fig. 1A). Bacterial survival rates were not affected at any concentrations of BS used in the experiment (Fig. 1B). On the other hand, nitrite accumulation at all time points (2, 24, and 48 h) was not affected in treatment cells without *B. abortus* infection (data not shown) but markedly increased in *Brucella-* infected BS-treated cells at 24 h (*p* < 0.05) pi in comparison with the control (Fig. 1C). NO is known to be an important effector molecule involved in the clearance of several intracellular bacteria including *B. abortus* [12]. Taken together, BS treatment did not directly affect the growth of *B. abortus* but slightly enhanced the production of nitrite during *Brucella* infection in macrophages.

### Effect of BS in *Brucella* Uptake and Intracellular Survival Within RAW264.7 Cells

**Fig. 2:**
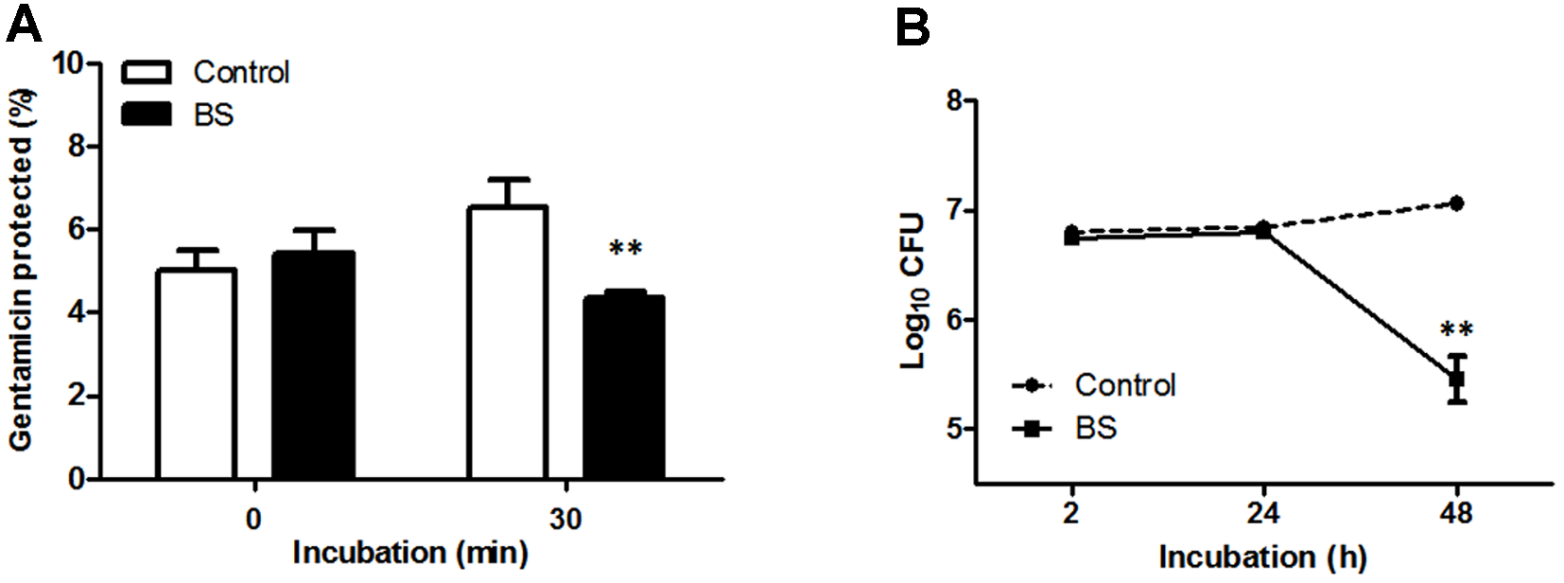
Effect of BS on internalization and intracellular survival of *B. abortus* in RAW264.7 cells. Cells were pre-incubated with or without BS for 4 h prior to infection with *B. abortus* for 0 and 30 min to determine (**A**) bacterial internalization efficiency. After 1 h infection with *B. abortus,* cells were incubated with or without BS for 2, 24, and 48 h to determine (**B**) bacterial intracellular survival efficiency. The data are presented as the means ± SD of triplicate experiments. **p < 0.01 *vs.* control group.

**Fig. 3:**
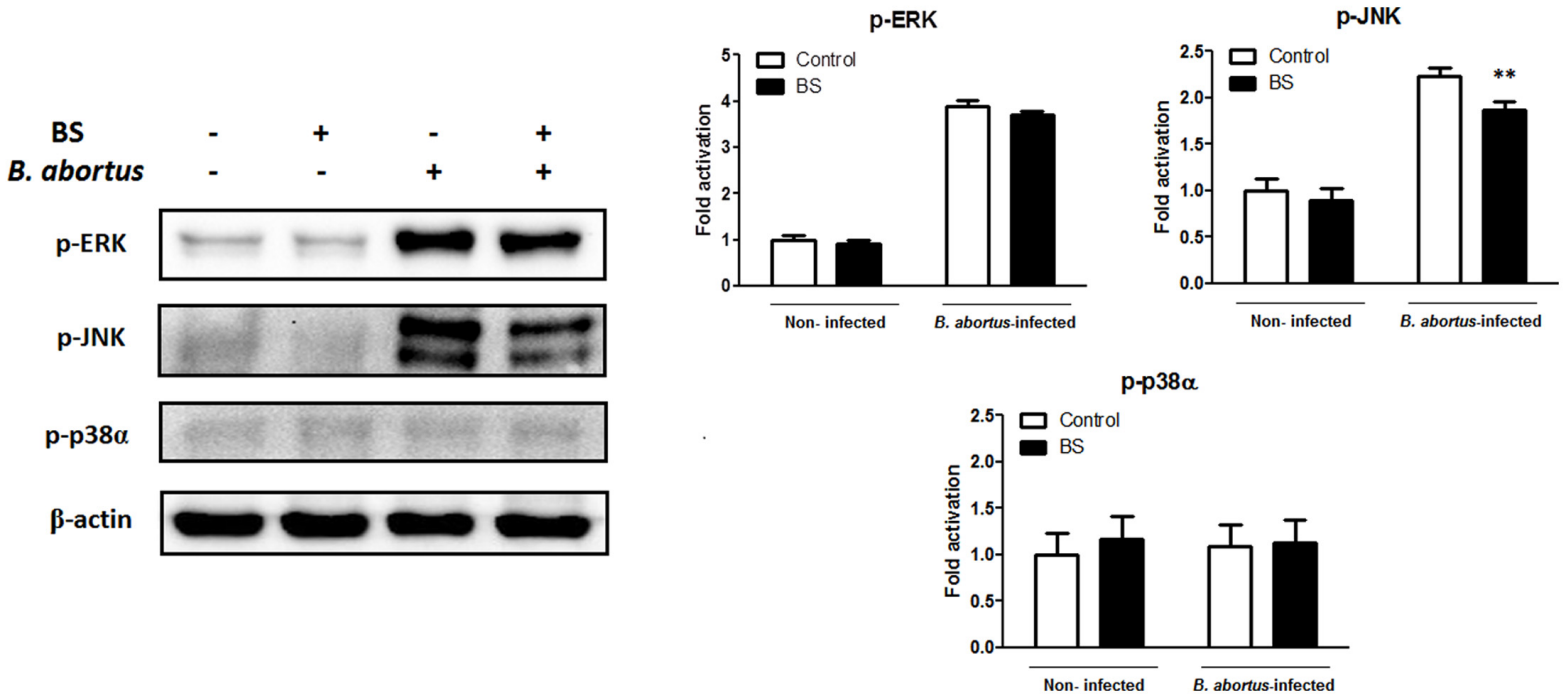
Effect of BS on MAPKs activation in RAW264.7 cells. Cells were pre-incubated with or without BS for 4h prior to infection with *B. abortus* for 30 min. Immunoblot analysis of total cell lysates was assessed using phospho-specific ERK1/2, JNK and p38α antibodies. The data are presented as the means ± SD of triplicate experiments. **p < 0.01 *vs.* control group.

Treatment of macrophages with BS markedly reduced the uptake of *B. abortus* into these cells at 30 min (*p* < 0.01) pi (Fig. 2A) and the bacterial intracellular survival at 48 h (*p* < 0.01) pi (Fig. 2B). Furthermore, we checked the phosphorylation level of mitogen-activated protein kinases (MAPKs) which are known to coordinately regulate gene expression, mitosis, metabolism, motility, survival, apoptosis and differentiation [13]. MAPKs also play an important role in the phagocytosis of bacteria hence a common target for obligate intracellular parasites for invasion. Phosphorylation of JNK was reduced (*p* < 0.01) in BS-treated cells while no differences were observed in the phosphorylation levels of ERK and p38α (Fig. 3). The inhibitory effect of BS on MAPKs could negatively affect the ability of *B. abortus* to invade and survive within macrophages.

### Effect of BS in Mice and Against *B. abortus* Infection

**Fig. 4:**
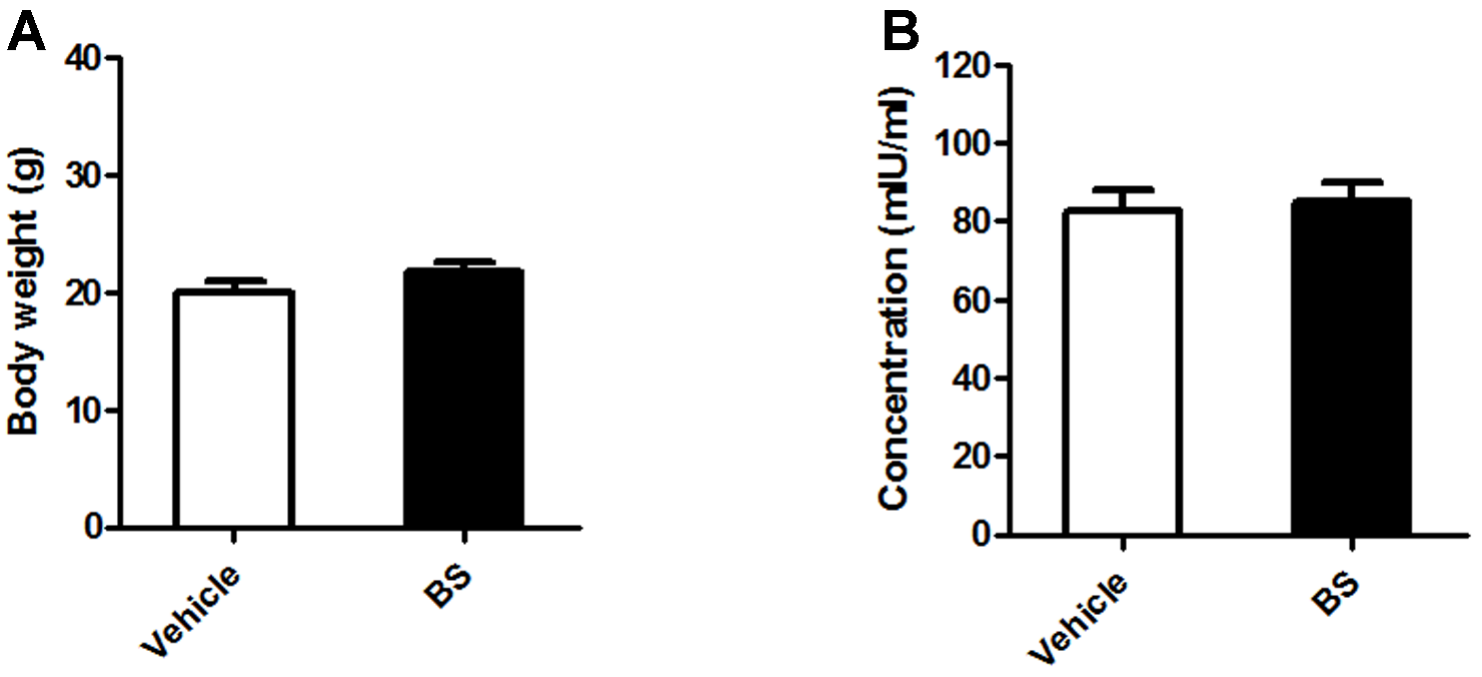
Effect of BS on body weight and serum ALT concentration in BALB/c mice. Oral treatment of BS or vehicle was done three days prior to infection and continued for 14 days. (**A**) Total body weight and (**B**) serum ALT concentration were determined at the end of the experiment. The data are presented as the means ± SD of five mice in each group.

**Fig. 5:**
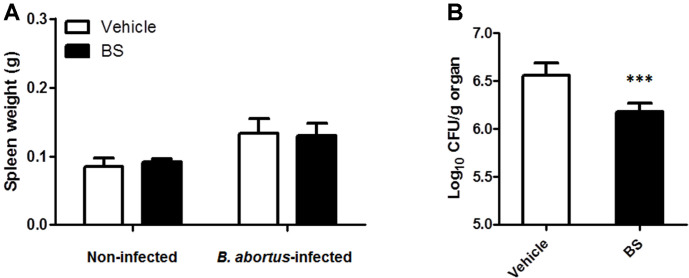
Effect of BS on splenic proliferation of *B. abortus* in BALB/c mice. At 14 days pi, (**A**) spleens were collected and weighed. A part of each spleen was homogenized, diluted in PBS, and plated onto Brucella agar to determine (**B**) the number of CFUs. The data are presented as the mean ± SD of five mice in each group. ***P < 0.001 *vs. Brucella-*infected vehicle control group.

No clinical symptoms during the entire treatment period were observed in all the animals. In the non-infected groups, we checked the effect of BS treatment in the total body weight and the serum ALT concentration. The results showed no differences in the total body weight (Fig. 4A) and serum ALT concentration (Fig. 4B), as well as in the splenic weight (Fig. 5A). On the other hand, since spleen is one of the most notable infected organs during *B. abortus* infection in mice with the higher number of CFU per gram of organ generally reaching its maximum CFU at two weeks pi [14], we checked the proliferation of *B. abortus* in these organs. First, no difference in the total weights of the spleens was observed between BS-treated (0.130 ± 0.017 g) and vehicle-treated mice (0.133 ± 0.021 g) (Fig. 5A), however a slight significant reduction (0.517; *p* < 0.01) in the number of log10 CFU was observed in mice treated with BS as compared to positive control group (Fig. 5B).

### Effect of BS on Cytokine Production In Mice

**Fig. 6:**
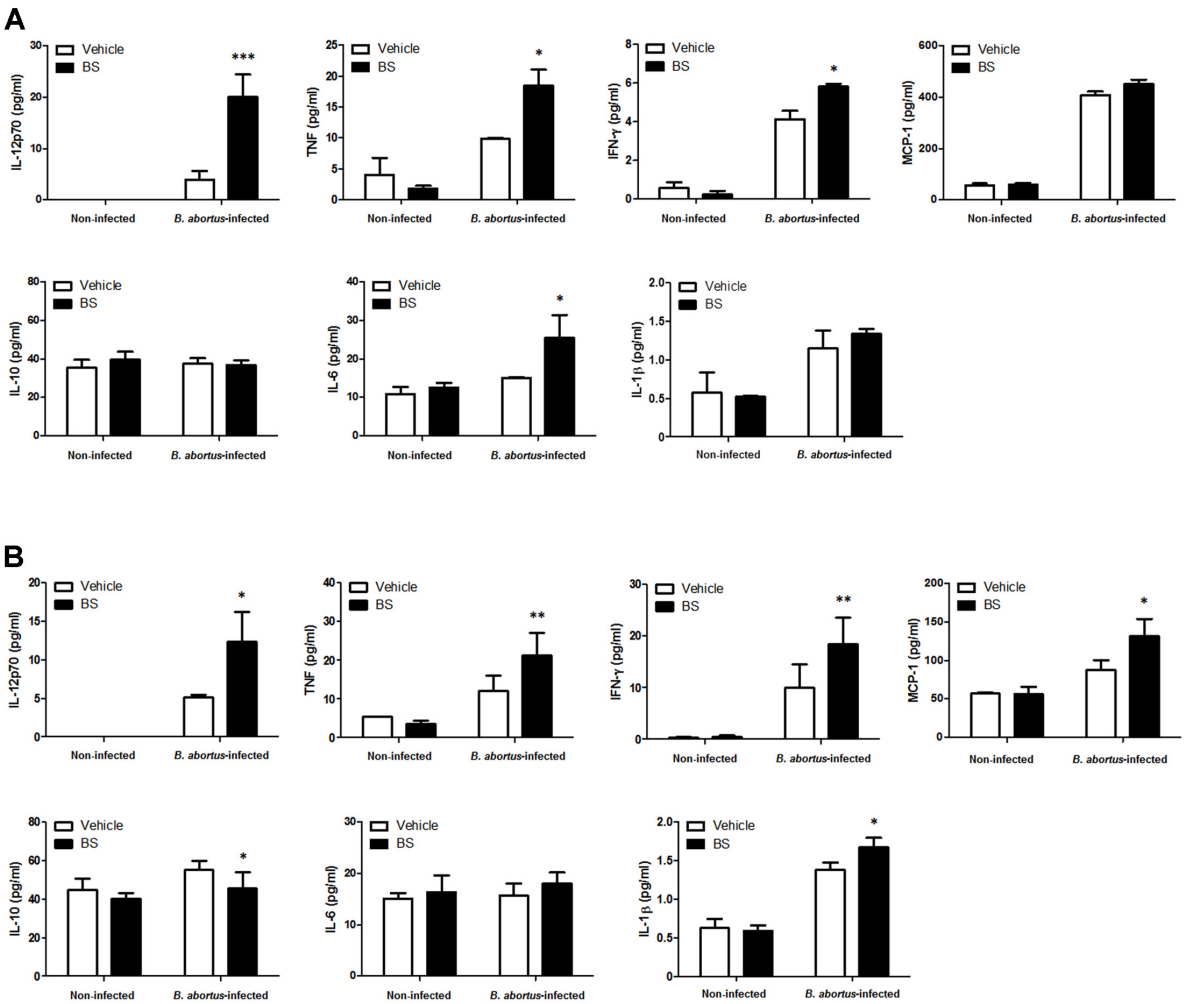
Effect of BS on production of serum cytokines in BALB/c mice. Blood samples were collected at 3 and 14 days pi. Cytokines from (**A**) non-infected and (**B**) *Brucella-*infected groups were measured from serum samples using CBA analysis. The data are presented as the mean ± SD of five mice in each group. *p < 0.05, **p < 0.01, and ***p < 0.001 *vs.* control group.

Serum samples collected at 3 and 14 days pi from all mice were evaluated for the potential immunoregulatory role of BS with or without *B. abortus* infection. In the *Brucella*-infected groups, significant increased levels of IL-12p70 (*p* < 0.001), TNF (*p* < 0.05) and IL-6 (*p* < 0.05) were observed at 3 days pi (Fig. 6A) while elevated levels of IL-12p70 (*p* < 0.05), TNF (*p* < 0.01), IFN-γ (*p* < 0.01), MCP-1 (*p* < 0.05) and IL-1β (*p* < 0.05) were observed at 14 days pi (Fig. 6B) in BS-treated mice as compared to control. Furthermore, reduced level of IL-10 (*p* < 0.05) was observed at 14 days pi (Fig. 6B). These findings indicate that BS treatment in mice could induce production of proinflammatory cytokines during *B. abortus* infection.

## Discussion

Brucellosis is one of the most widespread zoonotic diseases with a high morbidity in developing countries and yet still with undetermined optimal treatment [4]. Macrophages/monocytes are the primary target cells and a main reservoir of *Brucella* that constitute the first line of defense dedicated to eliminate invading microorganisms and display a wide array of phagocytic and inducible microbicidal functions such as the production of reactive oxygen and nitrogen intermediates through oxidative bursts [15]. However, *Brucella* use several strategies to survive within a hostile environment and cause persistent infection that are essential for the dissemination of the pathogens within the host [16]. After phagocytosis, it has been reported that over 90% of internalized *Brucella* in macrophages are killed but few still escape to establish an intracellular niche without affecting the survival of these cells [17]. In a study done by Boukes and Van de Venter [18], small but significant increases of 10-20% in phagocytosis of pHrodo *Escherichia coli* Bioparticles Conjugate and no significant effect on NO production were observed in human promonocytic U937 leukemia cells differentiated into monocyte-macrophages treated with BS. Although nitrite production was similar in non-infected BS-treated RAW264.7 cells, internalization of live *Brucella* was inhibited and nitrite production was slightly high during infection in these cells. The reduced number of surviving bacteria could be partly due to the presence of high nitrite accumulation since NO is known as a defense mechanism that accelerates the killing of intracellular *B. abortus* in RAW264.7 cells during the first 24 h of infection [19]. *Brucella* has the capacity to inhibit apoptosis and extend the life of infected phagocytic cells which contributes to its pathogenicity [20]. NO has a dual nature; it could be a cytotoxic effector molecule for invading microbes, viruses and parasites as well as to host cells themselves and neighboring cells that might interact with oxygen-derived radicals to generate molecules enhancing its cytotoxicity, and a vasodilator and hence potentially protective [21]. Macrophages are capable of sustained release of high NO levels that is initiated by inflammatory cytokines and bacterial products [21], hence it is possible that BS treatment in the current study activates macrophages to produce NO in order to control *Brucella* infection. Previously, we reported that internalization of *B. abortus* was inhibited in Korean red ginseng oil-treated RAW264.7 cells with reduced bacterial adherence, F-actin content and MAPK signaling protein phosphorylation [11]. Since phytochemical analysis of Korean red ginseng oil revealed that BS was one of its most abundant compounds, BS could possibly contribute in this inhibitory mechanism exhibited by Korean red ginseng oil treatment against *B. abortus* uptake in RAW264.7 cells although further investigations are required to verify these effects. In a study done by Loizou *et al*. [22], BS treatment was indicated to inhibit both vascular adhesion and intracellular adhesion molecule 1 expression in TNF-α-stimulated human aortic endothelial cells (HAECs). Fahy *et al*. [23] demonstrated a reduction in the uptake of beta-carotene in Caco-2 cells supplemented with BS at the same concentration used in our study. Furthermore, MAPKs are important mediators in many cellular functions including cytoskeletal rearrangement which has been a common and recurring target for obligate intracellular parasites for invasion [24]. Here BS treatment also led to downregulated MAPKs (JNK) which could interfere in the invasive mechanism of *Brucella* into macrophages. This is similar to suppression of phosphorylation of MAPKs (ERK, JNK, and p38α) by BS suggesting its regulatory role in preventing the growth of renal cancer in experimental animals [25].

BS possessed bactericidal activity against different bacterial species such as *Staphylococcus* (*S.*) *aureus, S. epidermidis, E. coli, Bacillus subtilis,* and *Enterococcus faecalis* [10]. However, in a study done by Lampronti *et al*. [26], BS did not interfere with the growth of *Pseudomonas* (*P.*) *aeruginosa* but reduce the *P. aeruginosa-*dependent expression of IL-8. This compound has also been reported to effectively protect mice from lethal infection caused by *Streptococcus pneumoniae,* which was not due to direct inhibition of bacterial growth but interaction with cholesterol-dependent toxin pneumolysin [27]. In the present study, BS did not directly inhibit the growth of *B. abortus* but treatment in mice slightly reduced the proliferation of these bacteria in the spleen possibly due to promotion of proinflammatory cytokine production since another contributing factor to the pathogenicity of *Brucella* is the induction of poor proinflammatory responses at the early stages of infection necessary to initiate host defense against microbial invasion [20, 28]. BS has been reported to exhibit anti-inflammatory and immune-modulating properties [29, 30]. Here we observed that elevated cytokines during *Brucella* infection in BS-treated mice including IL-12, TNF, IFN-γ, and IL-1β are important mediators of immune responses to intracellular pathogens [31, 32, 33], while MCP-1 also plays a central role in the maintenance of inflammation aimed to limit the infection [34]. On the other hand, reduced IL-10 production was also observed to help in the control of *Brucella.* IL-10 regulates the balance between the clearance of pathogen and the immune responses associated with the disease, and in a study done by Corsetti *et al.* [35], lack of endogenous IL-10 leads to *B. abortus* clearance in mice with enhanced production of proinflammatory cytokines. Furthermore, production of IL-10 is one of the causes of immunosuppression [36]. Oral treatment of BS in mice could reduce susceptibility to *B. abortus* infection possibly via enhanced release of proinflammatory cytokines. Furthermore, BS is structurally related to cholesterol that is slowly absorbed in the intestinal tract, hence other route of administration could be explored to maximize its beneficial effect. Overall, BS exhibited protective mechanisms against *B. abortus* infection via regulating invasion and intracellular survival of the pathogen, nitric oxide production and JNK phosphorylation in macrophages. On the other hand, BS slightly reduced bacterial splenic proliferation and differentially regulates cytokine production in mice. BS, as one of the most abundant compounds found in Korean red ginseng oil, could contribute to the inhibitory effects of Korean red ginseng oil against *B. abortus* infection encouraging further studies to establish practical use of BS as a supplement to feed additives, food products and medicines.
